# Serotype epidemiology and antibiotic resistance of pneumococcal isolates colonizing infants in Botswana (2016–2019)

**DOI:** 10.1371/journal.pone.0302400

**Published:** 2024-05-24

**Authors:** Jillian H. Hurst, Yazdani B. Shaik-Dasthagirisaheb, Loc Truong, Sefelani C. Boiditswe, Sweta M. Patel, Jodi Gilchrist, Julia Maciejewski, Kathy Luinstra, Marek Smieja, Andrew P. Steenhoff, Coleen K. Cunningham, Stephen I. Pelton, Matthew S. Kelly

**Affiliations:** 1 Division of Pediatric Infectious Diseases, Duke School of Medicine, Durham, North Carolina, United States of America; 2 Division of Pediatric Infectious Diseases, Boston Medical Center, Boston, Massachusetts, United States of America; 3 Botswana-University of Pennsylvania Partnership, Gaborone, Botswana; 4 Division of Pulmonary, Allergy and Critical Care Medicine, Duke University, Durham, North Carolina, United States of America; 5 Duke Global Health Institute, Duke University, Durham, North Carolina, United States of America; 6 Department of Laboratory Medicine, St. Joseph’s Healthcare, Hamilton, Ontario, Canada; 7 Department of Pathology and Molecular Medicine, McMaster University, Hamilton, Ontario, Canada; 8 Faculty of Health Sciences, Department of Pediatric and Adolescent Health, School of Medicine, University of Botswana, Gaborone, Botswana; 9 Department of Pediatrics, Division of Pediatric Infectious Diseases and Global Health Center, Children’s Hospital of Philadelphia, Philadelphia, Pennsylvania, United States of America; 10 Department of Pediatrics, University of California, Irvine, California, United States of America; 11 Children’s Hospital of Orange County, Orange, California, United States of America; 12 Division of Pediatric Infectious Diseases, Boston University Chobanian and Avedisian School of Medicine, Boston, Massachusetts, United States of America; Carnegie Mellon University, UNITED STATES

## Abstract

**Background:**

In 2012, Botswana introduced 13-valent pneumococcal conjugate vaccine (PCV-13) to its childhood immunization program in a 3+0 schedule, achieving coverage rates of above 90% by 2014. In other settings, PCV introduction has been followed by an increase in carriage or disease caused by non-vaccine serotypes, including some serotypes with a high prevalence of antibiotic resistance.

**Methods:**

We characterized the serotype epidemiology and antibiotic resistance of pneumococcal isolates cultured from nasopharyngeal samples collected from infants (≤12 months) in southeastern Botswana between 2016 and 2019. Capsular serotyping was performed using the Quellung reaction. E-tests were used to determine minimum inhibitory concentrations for common antibiotics.

**Results:**

We cultured 264 pneumococcal isolates from samples collected from 150 infants. At the time of sample collection, 81% of infants had received at least one dose of PCV-13 and 53% had completed the three-dose series. PCV-13 serotypes accounted for 27% of isolates, with the most prevalent vaccine serotypes being 19F (n = 20, 8%), 19A (n = 16, 6%), and 6A (n = 10, 4%). The most frequently identified non-vaccine serotypes were 23B (n = 29, 11%), 21 (n = 12, 5%), and 16F (n = 11, 4%). Only three (1%) pneumococcal isolates were resistant to amoxicillin; however, we observed an increasing prevalence of penicillin resistance using the meningitis breakpoint (2016: 41%, 2019: 71%; Cochran-Armitage test for trend, p = 0.0003) and non-susceptibility to trimethoprim-sulfamethoxazole (2016: 55%, 2019: 79%; p = 0.04). Three (1%) isolates were multi-drug resistant.

**Conclusions:**

PCV-13 serotypes accounted for a substantial proportion of isolates colonizing infants in Botswana during a four-year period starting four years after vaccine introduction. A low prevalence of amoxicillin resistance supports its continued use as the first-line agent for non-meningeal pneumococcal infections. The observed increase in penicillin resistance at the meningitis breakpoint and the low prevalence of resistance to ceftriaxone supports use of third-generation cephalosporins for empirical treatment of suspected bacterial meningitis.

## Introduction

*Streptococcus pneumoniae* (Spn; pneumococcus) is a major human pathogen that causes infections ranging from mild respiratory illnesses to invasive pneumococcal disease (IPD), which includes life-threatening infections such as bacteremia and meningitis [1]. Globally, Spn is a leading infectious cause of mortality, accounting for an estimated 829,000 deaths in 2019 [2]. The risk of mortality from Spn is highest during infancy and early childhood; among children less than five years of age, Spn causes more than 300,000 deaths each year, the majority of which occur in low- and middle-income countries (LMICs) [3]. Colonization of the upper respiratory tract by Spn is a prerequisite for the development of pneumococcal disease and horizontal transmission between individuals [4]. Prior studies have found that Spn carriage rates are highest among young children, with prevalence peaking in the second year of life [5]. Further, data from transmission studies indicate that infants and young children serve as the major reservoir for Spn, with regular contact with children increasing the likelihood of colonization and disease among adults [6,7]. Thus, Spn carriage among young children has a substantial impact on pneumococcal disease epidemiology across the lifespan.

Over the past two decades, the widespread adoption of pneumococcal conjugate vaccines (PCVs) has led to a dramatic reduction in the global burden of Spn disease, with child deaths from Spn declining by more than 50% during this time period [8,9]. Carriage of vaccine serotypes has significantly declined since the introduction of PCVs; however, this decline has consistently been accompanied by increased carriage of non-vaccine serotypes and, in many settings, increased disease caused by non-vaccine serotypes [10–13]. Moreover, as was observed prior to the development of PCVs, there is substantial heterogeneity in Spn serotype epidemiology and patterns of serotype replacement by geographical region, including among neighboring countries [14].

The shifts in serotype epidemiology that occur following PCV introduction can also affect the prevalence of antibiotic resistance among pneumococci. While implementation of PCVs has generally been associated with an initial reduction in the prevalence of antibiotic-resistant Spn isolates, several studies have documented increased resistance among specific vaccine and non-vaccine serotypes [12,15,16]. For example, the introduction of 7-valent pneumococcal conjugate vaccine (PCV-7) in the United States in 2000 resulted in a marked decline in carriage of vaccine serotypes that was accompanied by a decrease in penicillin-resistant isolates; however, two years after vaccine introduction, there was an abrupt increase in penicillin-resistant isolates driven primarily by the emergence of serotype 19A [17–19]. Further, more than half of pneumococci isolated from 7,156 children with non-invasive pneumococcal infections or IPD in the United States between 2011 and 2020 were resistant to at least one antibiotic, with a similar prevalence of antibiotic resistance observed in other geographical regions [15]. This rise in antibiotic resistance has the potential to lead to treatment failures, increased treatment costs, and higher morbidity and mortality among patients with pneumococcal infections [20]. Importantly, antibiotic resistance of pneumococci varies by region and country, even for isolates of the same serotype; thus, surveillance studies are critical to identify temporal changes in local serotype epidemiology and patterns of antibiotic resistance [12].

An understanding of local Spn epidemiology is necessary to effectively prevent and treat pneumococcal infections. Although most high-income countries have robust IPD surveillance programs, several barriers exist to establishing these programs in LMICs [21]. Because nasopharyngeal colonization precedes infections caused by Spn, carriage studies can be used to characterize local trends in serotype epidemiology, monitor and evaluate the impact of existing vaccine programs, and inform the deployment of new vaccines against Spn [1,22]. In the current study, we determined the serotype and antibiotic susceptibility of pneumococcal isolates colonizing infants in southeastern Botswana during a four-year period (2016–2019) beginning four years after the inclusion of 13-valent pneumococcal conjugate vaccine (PCV-13) in the national immunization program. We assessed for temporal changes in the serotype epidemiology and antibiotic resistance of circulating pneumococci during the study period and evaluated associations between infant characteristics and carriage of vaccine serotypes.

## Methods

### Study setting

Botswana is a landlocked country in southern Africa with a semi-arid climate and a rainy summer season that typically occurs from November to March. Gaborone is Botswana’s capital and largest city, with a population of 246,325 based on the 2022 census [23]. Botswana introduced PCV-13 (Prevnar-13; Pfizer) into its immunization program in July 2012. The vaccine is administered at 2, 3, and 4 months of age (3+0 schedule), and was introduced without a catch-up campaign. The estimated national coverage rate for the complete vaccine series in infants has been above 90% since 2014; between 2013–2014, the estimated rate of pneumococcal meningitis among children less than 5 years of age was 0.6 (95% confidence interval: 0.1–1.8) per 100,000 person years [24].

### Study population and procedures

This study included nasopharyngeal swabs and sociodemographic data from infants enrolled in a prospective cohort study of 300 mother-infant pairs conducted at a referral hospital and public clinics in or near Gaborone between 2016 and 2019, as described previously [25]. Nasopharyngeal samples were collected from infants monthly during the first six months of life and bimonthly thereafter until the infant was 12 months of age. Sample collection was independent of the presence of illness at the time of collection, and none of the children in this study developed IPD. Nasopharyngeal samples were placed directly into MSwab medium (Copan Italia, Brescia, Italy), transported to the National Health Laboratory in Gaborone, and frozen within 4 hours of collection to −80°C. All nasopharyngeal swab samples were tested for the presence of Spn using a quantitative PCR assay targeting the lytA gene, as previously described [26,27].

### Laboratory methods for pneumococcal serotype identification and characterization

A subset of nasopharyngeal samples from which Spn was detected by quantitative PCR were used for selective culture for pneumococci, including the first sample after birth from which Spn was detected for each infant; additional samples were selected for culture based on the *lytA* cycle threshold value to maximize the yield for isolation of pneumococci by culture. For broth enrichment cultures, frozen nasopharyngeal samples were thawed and mixed by vortexing. Approximately 200 μl of sample was added to 5 mL serum-supplemented Todd Hewitt Broth and incubated for 4 hours at 37ºC in 5% CO_2_. This enriched culture was plated on trypticase soy agar with 5% sheep blood supplemented with gentamicin and incubated for 18–24 hours at 37ºC in 5% CO_2_. Identification of Spn colonies was confirmed by α-hemolysis on these plates and inhibition by optochin. A representative colony was chosen from each culture and subjected to capsular serotyping. In the case where cultures appeared to contain multiple colony morphologies, additional colonies were chosen for further analysis. Capsular serotype identification was performed by Quellung reaction using serotype-specific pneumococcal antisera (SSI Diagnostica, Denmark) [28]. When multiple isolates of the same serotype were cultured from samples collected from the same infant, only the isolate from the earliest sample after birth was included in these analyses. Each cultured Spn isolate was tested for susceptibility to amoxicillin, azithromycin, ceftriaxone, penicillin, and trimethoprim-sulfamethoxazole (TMP-SMX) using E-tests (bioMérieux, Cambridge, MA). Spn cultures were collected from blood agar plates into tryptic soy broth and culture density was adjusted to the 0.5 McFarland Standard [29]. A swab of the bacterial solution was thoroughly streaked on a plate of Mueller Hinton agar with 5% sheep blood. Each antibiotic strip was gently placed on the bed of the media with sufficient room between strips to visualize zones of inhibition. Plates were incubated overnight at 37°C in 5% CO_2_. Minimum inhibitory concentrations (MICs) were determined as per manufacturer’s instructions. Strains were classified as susceptible, intermediate, or resistant using 2017 Clinical and Laboratory Standards Institute (CLSI) breakpoints [30].

### Statistical analyses

We classified serotypes/serogroups into PCV-13 serotypes (1, 3, 4, 5, 6A/C, 6B, 7F, 9V, 14, 18C, 19A, 19F, 23F), additional 15-valent pneumococcal conjugate vaccine (PCV-15) serotypes (22F, 33F), additional 20-valent pneumococcal conjugate vaccine (PCV-20) serotypes (8, 10A, 11A, 12F, 15B/C), and non-vaccine serotypes. Serotype 6C was included as a PCV-13 serotype due to cross-protection from the serotype 6A antigen [31]. We evaluated associations between infant and sample characteristics and isolate serotype classification using Chi-square or Fisher’s exact tests (categorical variables) and Wilcoxon rank-sum tests (continuous variables). We assessed for differences in the number of days since the last dose of PCV-13 among infants colonized with a PCV-13 serotype and a non-PCV-13 serotype using Welch’s t-test. We evaluated for changes in the prevalence of different serotype categories and antibiotic resistance over time using Cochran-Armitage tests for trend. We assessed for temporal changes in antibiotic minimum inhibitory concentrations (MICs) using Mann-Kendall trend tests. We used multivariable logistic regression to evaluate associations between infant characteristics and the carriage of PCV-13 serotypes. Statistical analyses were conducted using R version 4.3.0.

### Ethical statement

All study participants or their legal guardians provided written informed consent to participate in this study. The study protocol was approved by the Botswana Health Research and Development Committee, the Princess Marina Hospital ethics committee, and institutional review boards at the University of Pennsylvania, Children’s Hospital of Philadelphia, McMaster University, and Duke University.

## Results

### Participant characteristics

Of the 150 infants who had samples included in this study, slightly over half of the infants were female (n = 80, 53%), 48 (18%) were born to mothers living with HIV, and 14 were classified as low birth weight (birth weight <2500g; 5%) (Table 1). We cultured a total of 391 Spn isolates from samples collected from the 150 infants included in this study. When multiple isolates identified as a given serotype were cultured from samples collected from the same infant, only the isolate from the earliest sample after birth was included in subsequent analyses, resulting in a final set of 264 isolates cultured from 263 nasopharyngeal samples (two isolates of different serotypes were cultured from a single sample). The majority of Spn isolates (n = 213, 81%) were from infants who had received at least one dose of PCV-13, and more than half (n = 140, 53%) were from infants who had completed the three-dose vaccine series. Respiratory viruses were detected in 113 (43%) samples from which Spn was cultured, with rhinovirus/enterovirus being the most commonly identified virus (n = 76, 29%).

**Table 1:** Characteristics of infants and samples from which pneumococcal isolates included in this study were cultured.

**Infant characteristics (n = 150)**	**n (%)**
Female sex	80 (53%)
Maternal HIV infection	48 (32%)
Birth weight (g), median (IQR)	3122 (2859, 3435)
Low birth weight (<2500g)	14 (9%)
**Sample characteristics (n = 264**)	
Age (days), median (IQR)	155 (92, 299)
Breastfeeding	170 (64%)
Location of residence	
Rural	80 (30%)
Urban	184 (70%)
Year	
2016	51 (19%)
2017	92 (35%)
2018	93 (35%)
2019	28 (11%)
Season	
Dry	190 (72%)
Rainy	74 (28%)
Doses of PCV-13 vaccine received	
0	51 (19%)
1	44 (17%)
2	29 (11%)
3	140 (53%)
Antibiotic exposure since prior study visit	58 (22%)
Amoxicillin	28 (11%)
Metronidazole	6 (2%)
Trimethoprim-sulfamethoxazole	19 (7%)
Respiratory virus detection	113 (43%)
Adenovirus	11 (4%)
Rhinovirus/enterovirus	76 (29%)
Other	26 (10%)

g, grams; IQR, interquartile range.

### Serotype epidemiology of pneumococcal isolates

**Fig 1:**
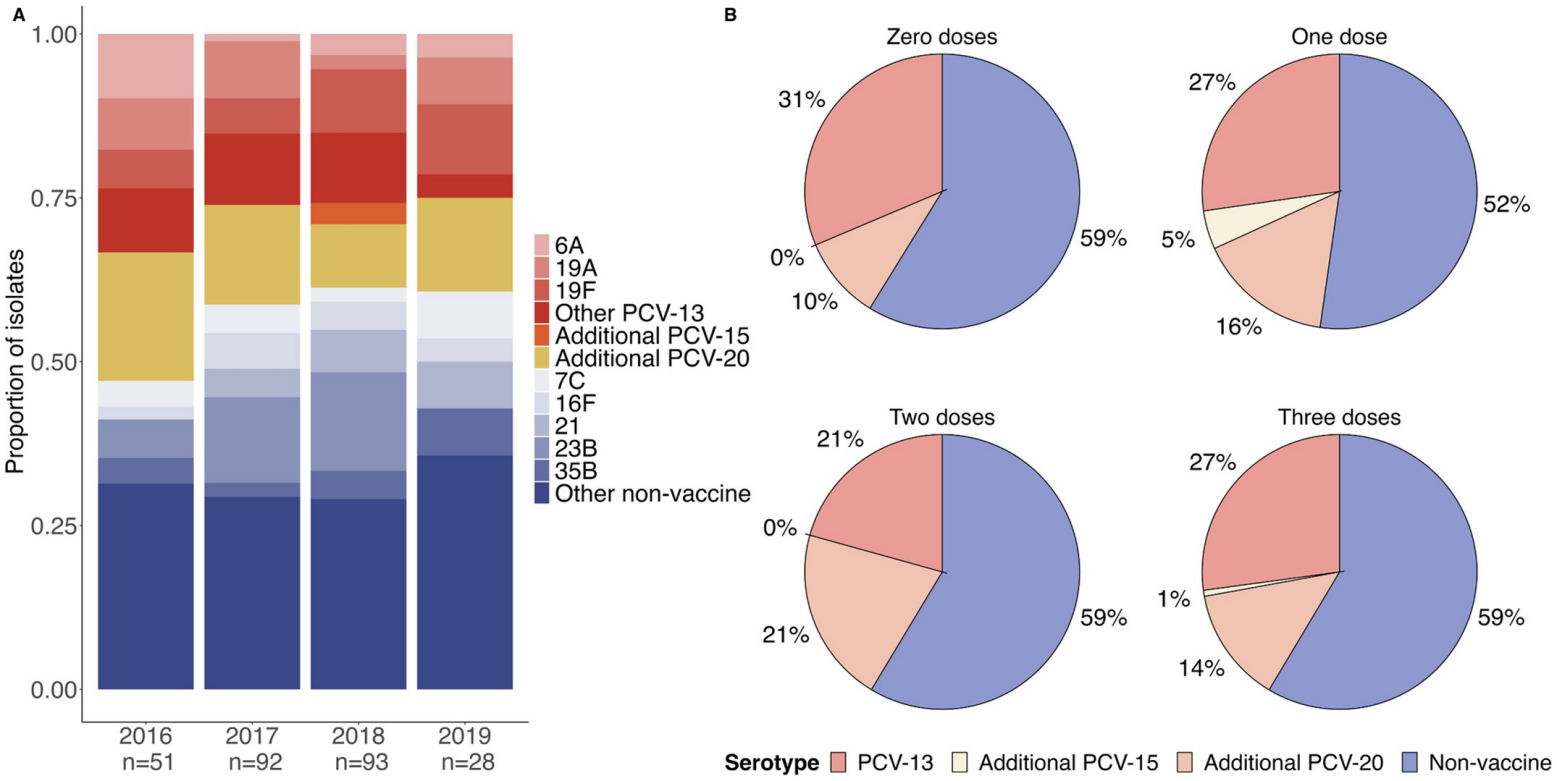
Prevalence of pneumococcal serotypes by year and by number of PCV-13 doses received. Pneumococcal isolates from infant nasopharyngeal swabs collected during the first year of life were subjected to the Quellung reaction to identify specific serotypes. A) Bars depict the proportion of specific serotypes of pneumococcal isolates collected from infants in Botswana by year of enrollment. PCV-13 serotypes are shaded red to pink, additional serotypes covered by PCV-15 are in orange, additional serotypes covered by PCV-20 are shaded in yellow, and non-vaccine serotypes are presented in shades of blue. B) Each pie chart shows the proportion of isolates identified as PCV-13 serotypes, additional serotypes covered by PCV-15 and PCV-20, and non-vaccine serotypes in infants who had received zero, one, two, or three doses of PCV-13 at the time of sampling.

Seventy-two of 264 (27%) Spn isolates were serotypes contained within PCV-13 (Fig 1), with the most common vaccine serotypes being 19F (n = 20, 8%), 19A (n = 16, 6%), and 6A (n = 10, 4%) (S1 Table). Only three (<1%) isolates were identified as the additional serotypes contained within PCV-15 (22F, 33F), while 37 (14%) isolates were additional serotypes contained in PCV-20 but not PCV-15, with the most common additional PCV-20 serotypes being 15B (n = 15, 6%) and 11A (n = 14, 5%). One hundred fifty-two (58%) Spn isolates were identified as serotypes not contained within any currently available conjugate vaccine, with the most prevalent non-vaccine serotypes being 23B (n = 29, 11%), 21 (n = 12, 5%), 16F (n = 11, 4%), 7C (n = 10, 4%), and 35B (n = 10, 4%). The proportion of isolates classified as PCV-13 (Cochrane-Armitage test; p = 0.39), additional PCV-15 (p = 0.23), additional PCV-20 (p = 0.19), or non-vaccine (p = 0.15) serotypes did not change during the study period. Moreover, the Spn colonization density did not differ between PCV-13 serotypes and other serotype categories (median of 7.45 vs. 7.52 log_10_ copies/mL; Wilcoxon rank-sum test, p = 0.39).

### Infant characteristics associated with PCV-13 serotype carriage

We next evaluated carriage of vaccine serotypes by the number of doses of PCV-13 received at the time of sample collection (Fig 1B). The proportions of isolates classified as PCV-13, additional PCV-15, additional PCV-20, or non-vaccine serotypes did not differ based on vaccination status (Chi-square test, p = 0.71). Among infants who had received three doses of PCV-13, 38 (27%) Spn isolates were classified as PCV-13 serotypes. There was no difference in the time since receipt of the most recent dose of PCV-13 among infants colonized with PCV-13 serotypes and infants with non-PCV-13 serotypes [mean (standard deviation): 104 (82) days vs. 107 (79) days; Welch’s t- test, p = 0.82]. We additionally sought to identify infant sociodemographic and clinical characteristics associated with PCV-13 serotype carriage. At the time of sample collection, the characteristics of infants from whom a PCV-13 serotype was cultured were similar to those of infants colonized by a non-PCV-13 serotype (Table 2). Specifically, these groups did not differ based on sex, maternal HIV status, breastfeeding status, birth weight, location of residence, PCV-13 doses received, recent receipt of antibiotics, or respiratory virus detection. Further, the likelihood of detecting a PCV-13 serotype isolate did not differ by collection year or season of sample collection.

**Table 2:** Multivariable logistic regression analysis of infant characteristics associated with PCV-13 serotype carriage.

Characteristics	PCV-13 Serotype (n = 72, 27%)	Non-PCV-13 Serotype (n = 192, 73%)	Odds Ratio (95% CI)	p
Age (days), median (IQR)	158 (90, 299)	154 (93, 298)	1.00 (0.99, 1.00)	0.80
Male sex	36 (50%)	100 (52%)	1.05 (0.59, 1.87)	0.86
Maternal HIV infection	23 (25%)	49 (28%)	0.88 (0.35, 2.18)	0.79
Breastfeeding	48 (67%)	123 (64%)	1.10 (0.44, 2.82)	0.84
Birth weight (g), median (IQR)	3210 (2795, 3441)	3128 (2910, 3440)	1.00 (0.99, 1.00)	0.85
Low birth weight (<2500g)	5 (7%)	19 (10%)	0.67 (0.17, 2.31)	0.54
Location of residence				0.33
Rural	19 (26%)	61 (31%)	Reference	
Urban	53 (74%)	131 (68%)	1.36 (0.74, 2.59)	
Year	—	—	0.84 (0.60, 1.15)	0.29
2016	17 (24%)	34 (18%)		
2017	24 (33%)	68 (35%)		
2018	24 (33%)	69 (36%)		
2019	7 (10%)	21 (11%)		
Season				0.94
Dry	52 (72%)	138 (72%)	Reference	
Rainy	20 (28%)	54 (28%)	1.02 (0.54, 1.88)	
Doses of PCV-13 vaccine received, median (IQR)	3 (1, 3)	3 (1, 3)	0.93 (0.61, 1.41)	0.75
Antibiotic exposure since prior study visit	16 (22%)	42 (22%)	1.02 (0.50, 2.00)	0.96
Amoxicillin	10 (14%)	18 (9%)		
Metronidazole	1 (1%)	5 (3%)		
Trimethoprim-sulfamethoxazole	6 (8%)	13 (7%)		
Respiratory virus detection	31 (43%)	82 (43%)	1.00 (0.57, 1.74)	0.99
Adenovirus	2 (3%)	9 (5%)		
Enterovirus/Rhinovirus	23 (32%)	53 (28%)		
Other	6 (10%)	20 (8%)		

CI, confidence interval; IQR, interquartile range; g, grams.

### Antibiotic susceptibility of pneumococcal isolates

**Fig 2:**
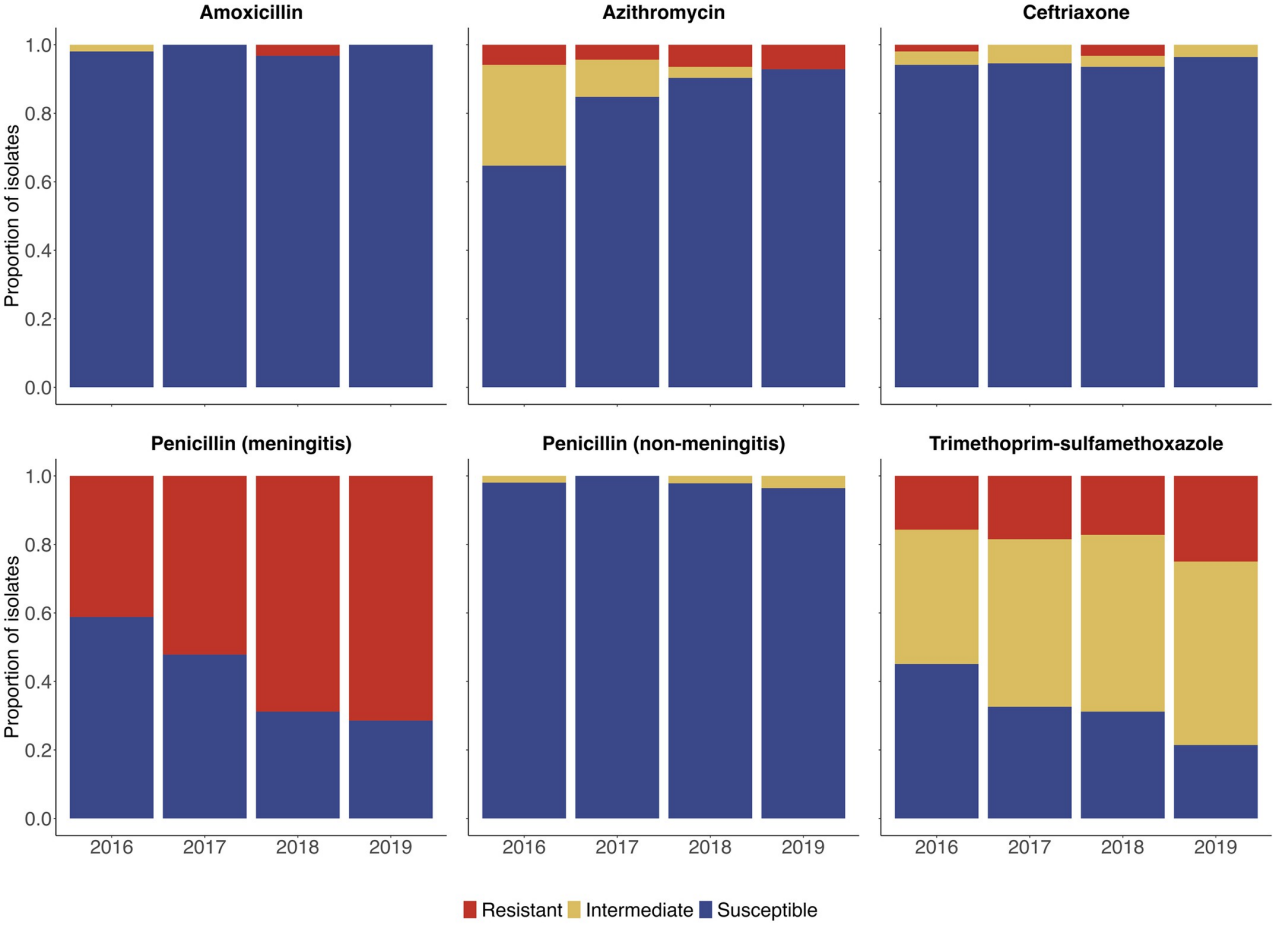
Proportion of pneumococcal isolates classified as susceptible, intermediate, or resistant to specific antibiotics by year. E-tests were used to determine the minimum inhibitory concentrations for pneumococci to amoxicillin, azithromycin, ceftriaxone, penicillin, and trimethoprim-sulfamethoxazole. Antibiotic susceptibility classifications were determined using the 2017 Clinical & Laboratory Standards Institute (CLSI) breakpoints.

Of 264 Spn isolates, 59 (22%) were resistant to at least one antibiotic, with the prevalence of antibiotic-resistant isolates being stable over the time period of the study (18% in 2016, 21% in 2017, 25% in 2018, and 29% in 2019; Cochrane-Armitage test for trend, p = 0.19). Spn isolates were most frequently resistant to TMP-SMX (n = 48, 18%), including more than one-quarter of isolates identified as serotypes 15A, 19A, 23A, and 23B (S2 Table, Fig 2). Relatively few isolates were resistant to amoxicillin (n = 3, 1%), azithromycin (n = 15, 6%), or ceftriaxone (n = 4, 2%). We did not identify any Spn isolates that were resistant to penicillin at the non-meningitis breakpoint; however, more than half of isolates were resistant to penicillin at the meningitis breakpoint (n = 152, 58%), including over 90% of isolates identified as serotypes 19A, 19F, and 35B (S2 Table, Fig 2). The proportion of isolates that were resistant to penicillin at the meningitis breakpoint increased over the course of the study (41% in 2016, 52% in 2017, 69% in 2018, and 71% in 2019; Cochrane-Armitage test for trend, p = 0.0003), a trend that was observed among both PCV-13 (p = 0.03) and non-PCV-13 (p = 0.002) serotypes. Finally, we identified three multidrug-resistant Spn isolates: one 19A isolate (resistant to azithromycin, ceftriaxone, and TMP-SMX), one 19F isolate (resistant to amoxicillin, azithromycin, and TMP-SMX), and one 35B isolate (resistant to amoxicillin, azithromycin, and TMP-SMX).

**Fig 3:**
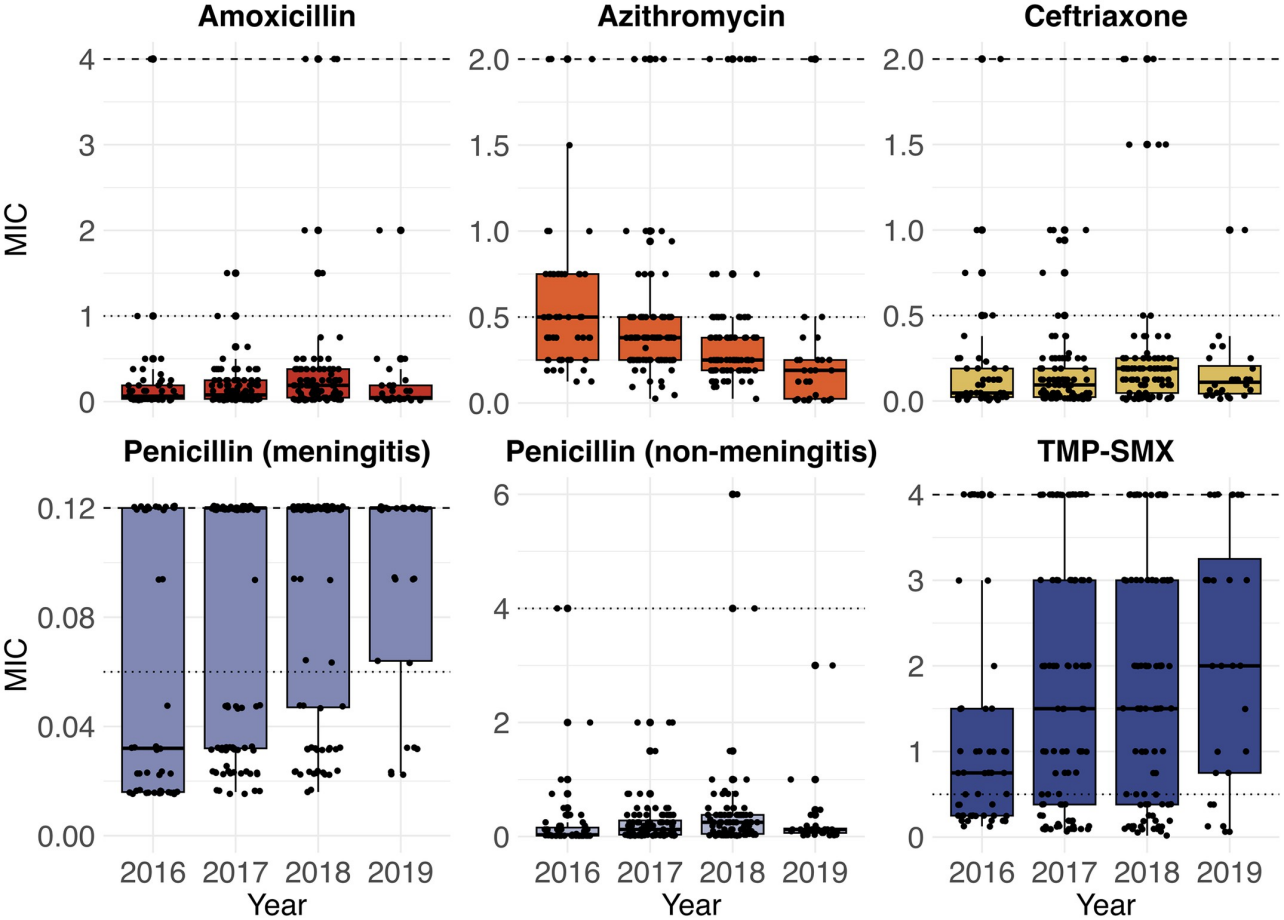
Minimum inhibitory concentrations (MICs) of pneumococcal isolates for common antibiotics by year. E-tests were used to determine the MICs for pneumococci to amoxicillin, azithromycin, ceftriaxone, penicillin, and trimethoprim-sulfamethoxazole (TMP-SMX). Each point represents the MIC for a single pneumococcal isolate. The breakpoints for intermediate (dotted line) and resistant (dashed line) are based on the 2017 Clinical & Laboratory Standards Institute (CLSI) breakpoints. All isolates with MICs above the breakpoint for resistance are shown at the dashed line.

Although the prevalence of antibiotic resistance was relatively low among pneumococci in this study, 197 (75%) isolates were non-susceptible to at least one antibiotic (S3 Table). Relatively few Spn isolates were non-susceptible to amoxicillin (n = 4, 2%), azithromycin (n = 43, 16%), ceftriaxone (n = 15, 6%), or penicillin at the non-meningitis breakpoint (n = 4, 2%). However, 176 (76%) isolates were non-susceptible to TMP-SMX, including 54 (75%) of the isolates identified as PCV-13 serotypes and 122 (64%) of the isolates identified as non-PCV-13 serotypes. The proportion of Spn isolates that were non-susceptible to TMP-SMX increased during the study (55% in 2016, 67% in 2017, 69% in 2018, and 79% in 2019; Cochrane-Armitage test for trend, p = 0.04); however, this trend was only observed among PCV-13 serotype isolates (p = 0.03). In contrast, non-susceptibility to azithromycin decreased over time among all isolates (p = 0.0001) and isolates identified as non-PCV-13 serotypes (p<0.0001). No significant trends in the values of MICs for specific antibiotics were observed over time during the study period (Fig 3, S4 Table).

## Discussion

We found that PCV-13 serotype carriage was stable over a four-year period among infants in Botswana four years after introduction of the vaccine into the national immunization program. Notably, PCV-13 serotype carriage did not differ by vaccination status or other recorded infant characteristics. Twenty-two percent of Spn isolates were resistant to at least one antibiotic with the prevalence of antibiotic resistance among isolates remaining stable during the study period. However, we observed increases in the proportions of Spn isolates that were resistant to penicillin at the meningitis breakpoint and isolates that were non-susceptible to TMP-SMX. Our findings provide valuable information regarding the serotype epidemiology and antibiotic resistance of Spn following PCV-13 introduction in Botswana, a country with a high burden of pneumococcal disease that lacks a robust IPD surveillance program [32].

We observed that PCV-13 serotype carriage was stable during the study period, with vaccine serotypes making up 27% of Spn isolates. These findings are consistent with previous studies conducted in southern Africa that reported that PCV-13 serotypes continued to account for approximately one-quarter of Spn isolates following vaccine introduction [33–35]. We additionally observed that the number of vaccine doses received by infants was not associated with PCV-13 serotype carriage. This observation that the prevalence of PCV-13 carriage did not differ based on vaccination status, combined with our prior data demonstrating a marked decline in PCV-13 serotype carriage after vaccine introduction in Botswana, suggest that a sufficient portion of the population is vaccinated to provide herd protection to children who are not fully vaccinated [33,36]. Botswana currently uses a 3+0 schedule for PCV-13 administration, with infants receiving doses at approximately 2, 3, and 4 months of age. Recent studies have indicated that this schedule may be associated with continued circulation of vaccine serotypes and breakthrough IPD infections among older children, likely due to antibody waning in the year following the third dose [37]. These observations have led several countries to implement PCV schedules with a booster dose in the second year of life, potentially reducing the reservoir of vaccine serotypes in older children [9]. We found that PCV-13 serotype carriage did not differ based on the duration of time from the last PCV-13 dose; however, antibody waning may be more pronounced among children older than one year of age.

We specifically identified continued high prevalence of the PCV-13 serotypes 19A and 19F [33]. Recent surveillance studies conducted in the United Kingdom and The Gambia similarly identified 19A and 19F as serotypes that persisted after PCV-13 introduction [38,39]. The continued circulation of these serotypes is concerning as serogroup 19 has been identified as a common cause of IPD among children in several settings, including Brazil, South Africa, and Australia, and serotype 19F has a high prevalence of antibiotic resistance [40–42]. The mechanisms underlying such persistence of vaccine serotypes are not well understood; however, advances in genomic sequencing technologies may provide new insights. For instance, a recent analysis of serogroup 19 isolates from children with IPD in Australia identified core genome sequence clusters that emerged after PCV-7 and PCV-13 introduction and that were associated with antibiotic resistance and vaccine failure [40]. Additionally, a genomic analysis of serotype 19A isolates from IPD cases in Ireland identified a sub-clade of 19A that was associated with vaccine failure and characterized by a specific allele of the *galE* gene that may contribute to pathogenicity [43]. Future studies are needed to identify genomic markers that influence serotype persistence and vaccine failure in populations with varied exposure patterns and selective pressures.

In many settings, the introduction of PCVs has been accompanied by an increase in the prevalence of both non-vaccine serotype carriage and IPD [12,44,45]. While our study only evaluated carriage and was not powered to detect changes in the carriage of individual serotypes, the non-vaccine serotypes 7C, 16F, 21, 23B, and 35B were highly prevalent in the study population. Many of these non-vaccine serotypes have been observed to increase following PCV-13 introduction in other settings [38,39,46,47]. Moreover, several of these serotypes are common causes of IPD or have a high prevalence of antibiotic resistance [47–49]. Taken together, our findings and those of these prior studies demonstrate that continued surveillance is needed to identify replacement serotypes for both carriage and IPD and to identify population characteristics associated with patterns of serotype replacement.

Implementation of PCVs has also been associated with changes in the prevalence of antibiotic resistance among both vaccine and non-vaccine serotypes [50]. In our cohort, resistance to commonly used antibiotics was generally low, although more than three-quarters of isolates were non-susceptible to TMP-SMX. TMP-SMX is often used for the treatment of mild respiratory and gastrointestinal infections among young children in Botswana, and the rising prevalence of isolates non-susceptible to TMP-SMX could reflect increasing use of this antibiotic in local pediatric populations. Alternatively, TMP-SMX has been used widely in Botswana as an agent for *Pneumocystis jirovecii* pneumonia prophylaxis among people living with HIV, which may have promoted selection of pneumococci with some degree of resistance to this antibiotic [51]. We additionally observed a high and increasing prevalence of resistance to penicillin at the meningitis breakpoint. Given that Spn accounts for approximately 20% of meningitis cases among children in Botswana, the observed resistance to penicillin at the meningitis breakpoint supports use of third-generation cephalosporins as the first-line treatment for suspected bacterial meningitis among children in this setting [24].

This study has a number of strengths and limitations. Strengths include the relatively large sample size, the high PCV-13 coverage rate in Botswana, and the evaluation of Spn isolates across a four-year period after vaccine introduction. Limitations include analyses being limited to Spn isolates that could be cultured; given that prior studies suggest that colonization density may vary by serotype, this approach could have resulted in oversampling of serotypes that colonize the nasopharynx at high density [52]. Additionally, study subjects were recruited at four sites in or near Gaborone, Botswana, which may limit the generalizability of these findings to other geographical areas. The study included only Spn isolates from infants, thus precluding analyses of pneumococcal carriage in other age groups. Finally, data on the serotypes that caused IPD in Botswana during the study period were unavailable, and future studies are needed to determine the serotype epidemiology and antibiotic resistance of isolates that cause pneumococcal disease in the country.

In summary, our findings indicate that PCV-13 implementation resulted in sustained reductions in carriage of vaccine serotypes among infants in Botswana. We additionally determined the prevalence of antibiotic resistance among pneumococci colonizing infants in this setting, providing valuable local data to inform the treatment of children with suspected pneumococcal infections.

## Supporting information

S1 Checklist (DOCX)

S1 Table Pneumococcal serotype prevalence by vaccine valency and collection year. (DOCX)

S2 Table Prevalence of antibiotic resistant isolates by pneumococcal serotype. (DOCX)

S3 Table Prevalence of antibiotic non-susceptible isolates by pneumococcal serotype. (DOCX)

S4 Table Distribution of minimum inhibitory concentrations to specific antibiotics among pneumococcal isolates by year. (DOCX)
