# Supplementary material for: Serotype epidemiology and antibiotic resistance of pneumococcal isolates colonizing infants in Botswana (2016–2019)
Source: PLoS One. 2024 May 24;19(5):e0302400. doi: 10.1371/journal.pone.0302400 (PMC11125537; doi:10.1371/journal.pone.0302400)
Supplement: S1 Table — (DOCX) [file pone.0302400.s002.docx]

| **Supplemental Table 1.** Pneumococcal serotype prevalence by vaccine valency and collection year | | | | | |  |
| --- | --- | --- | --- | --- | --- | --- |
| **Serotypes** | | **Total**  **(n=264)** | **2016**  **(n=51)** | **2017**  **(n=92)** | **2018**  **(n=93)** | **2019**  **(n=28)** |
| **PCV-13** | | **72 (27%)** | **17 (33%)** | **24 (26%)** | **24 (26%)** | **7 (25%)** |
|  | 1 | 0 (0%) | 0 (0%) | 0 (0%) | 0 (0%) | 0 (0%) |
|  | 3 | 5 (2%) | 1 (2%) | 1 (1%) | 3 (3%) | 0 (0%) |
|  | 4 | 1 (1%) | 0 (0%) | 1 (1%) | 0 (0%) | 0 (0%) |
|  | 5 | 0 (0%) | 0 (0%) | 0 (0%) | 0 (0%) | 0 (0%) |
|  | 6A | 10 (4%) | 5 (10%) | 1 (1%) | 3 (3%) | 1 (4%) |
|  | 6B | 2 (1%) | 0 (0%) | 1 (1%) | 1 (1%) | 0 (0%) |
|  | 6C* | 3 (1%) | 0 (0%) | 1 (1%) | 2 (2%) | 0 (0%) |
|  | 7F | 1 (1%) | 1 (1%) | 0 (0%) | 0 (0%) | 0 (0%) |
|  | 9V | 2 (1%) | 2 (4%) | 0 (0%) | 0 (0%) | 0 (0%) |
|  | 14 | 2 (1%) | 0 (0%) | 2 (2%) | 0 (0%) | 0 (0%) |
|  | 18C | 3 (1%) | 1 (2%) | 1 (1%) | 0 (0%) | 1 (4%) |
|  | 19A | 16 (6%) | 4 (8%) | 8 (9%) | 2 (2%) | 2 (7%) |
|  | 19F | 20 (8%) | 3 (6%) | 5 (5%) | 9 (10%) | 3 (11%) |
|  | 23F | 7 (3%) | 0 (0%) | 3 (3%) | 4 (4%) | 0 (0%) |
| **Additional PCV-15** | | **3 (1%)** | **0 (0%)** | **0 (0%)** | **3 (3%)** | **0 (0%)** |
|  | 22F | 1 (1%) | 0 (0%) | 0 (0%) | 1 (1%) | 0 (0%) |
|  | 33F | 2 (1%) | 0 (0%) | 0 (0%) | 2 (2%) | 0 (0%) |
| **Additional PCV-20** | | **37 (14%)** | **10 (20%)** | **14 (15%)** | **9 (10%)** | **4 (14%)** |
|  | 11A | 14 (5%) | 4 (8%) | 6 (7%) | 2 (2%) | 2 (7%) |
|  | 15B | 15 (6%) | 4 (8%) | 4 (4%) | 6 (6%) | 1 (4%) |
|  | Other | 8 (3%) | 2 (4%) | 4 (4%) | 1 (1%) | 1 (4%) |
| **Non-vaccine** | | **152 (58%)** | **24 (47%)** | **57 (60%)** | **61 (62%)** | **17 (61%)** |
|  | 7C | 10 (4%) | 2 (4%) | 4 (4%) | 2 (2%) | 2 (7%) |
|  | 9N | 5 (2%) | 1 (2%) | 1 (1%) | 2 (2%) | 1 (4%) |
|  | 15A | 9 (3%) | 1 (2%) | 5 (5%) | 2 (2%) | 1 (4%) |
|  | 15C | 6 (2%) | 0 (0%) | 4 (4%) | 1 (1%) | 1 (4%) |
|  | 16F | 11 (4%) | 1 (2%) | 5 (5%) | 4 (4%) | 1 (4%) |
|  | 17F | 7 (3%) | 2 (4%) | 1 (1%) | 2 (2%) | 2 (7%) |
|  | 21 | 12 (5%) | 0 (0%) | 4 (4%) | 6 (6%) | 2 (7%) |
|  | 23A | 7 (3%) | 2 (4%) | 1 (1%) | 2 (2%) | 2 (7%) |
|  | 23B | 29 (11%) | 3 (6%) | 12 (13%) | 14 (15%) | 0 (0%) |
|  | 34 | 8 (3%) | 2 (4%) | 2 (2%) | 2 (2%) | 2 (7%) |
|  | 35B | 10 (4%) | 2 (4%) | 2 (2%) | 4 (4%) | 2 (7%) |
|  | 35F | 9 (3%) | 3 (6%) | 2 (2%) | 3 (3%) | 1 (4%) |
|  | Other | 29 (11%) | 5 (10%) | 11 (12%) | 13 (14%) | 0 (0%) |
| ***Serotype 6C is included as a PCV-13 serotype due to cross-protection provided by serotype 6A | | | | | |  |
