# Supplementary material for: Serotype epidemiology and antibiotic resistance of pneumococcal isolates colonizing infants in Botswana (2016–2019)
Source: PLoS One. 2024 May 24;19(5):e0302400. doi: 10.1371/journal.pone.0302400 (PMC11125537; doi:10.1371/journal.pone.0302400)
Supplement: S3 Table — (DOCX) [file pone.0302400.s004.docx]

| **Supplemental Table 3.** Prevalence of antibiotic non-susceptible isolates by pneumococcal serotype | | | | | | | |
| --- | --- | --- | --- | --- | --- | --- | --- |
| **Serotypes** | | **Amoxicillin** | **Azithromycin** | **Ceftriaxone** | **Penicillin**  **(meningitis)** | **Penicillin**  **(non-meningitis)** | **TMP-SMX** |
| **All (n=264)** | | **4 (2%)** | **43 (16%)** | **15 (6%)** | **157 (59%)** | **4 (2%)** | **176 (67%)** |
| **PCV-13 (n=72)** | | **3 (4%)** | **19 (26%)** | **6 (8%)** | **48 (67%)** | **3 (4%)** | **54 (75%)** |
|  | 3 (n=5) | 0 (0%) | 0 (0%) | 0 (0%) | 1 (20%) | 0 (0%) | 1 (20%) |
|  | 4 (n=1) | 0 (0%) | 0 (0%) | 0 (0%) | 1 (100%) | 0 (0%) | 1 (100%) |
|  | 6A (n=10) | 1 (10%) | 1 (10%) | 0 (0%) | 2 (20%) | 0 (0%) | 7 (70%) |
|  | 6B (n=2) | 0 (0%) | 1 (50%) | 0 (0%) | 2 (100%) | 0 (0%) | 2 (100%) |
|  | 6C* (n=3) | 0 (0%) | 0 (0%) | 0 (0%) | 2 (67%) | 0 (0%) | 3 (100%) |
|  | 14 (n=2) | 0 (0%) | 1 (50%) | 0 (0%) | 1 (50%) | 0 (0%) | 1 (50%) |
|  | 18C (n=3) | 0 (0%) | 2 (67%) | 0 (0%) | 0 (0%) | 0 (0%) | 2 (67%) |
|  | 19A (n=16) | 0 (0%) | 3 (19%) | 2 (13%) | 15 (94%) | 0 (0%) | 14 (88%) |
|  | 19F (n=20) | 2 (2%) | 9 (45%) | 4 (20%) | 19 (95%) | 3 (15%) | 19 (95%) |
|  | 23F (n=7) | 0 (0%) | 2 (29%) | 0 (0%) | 5 (71%) | 0 (0%) | 4 (57%) |
|  | Other (n=26) | 0 (0%) | 0 (0%) | 0 (0%) | 12 (46%) | 0 (0%) | 0 (0%) |
| **Additional PCV-15 (n=3)** | | **0 (0%)** | **0 (0%)** | **0 (0%)** | **1 (33%)** | **0 (0%)** | **2 (67%)** |
| **Additional PCV-20 (n=37)** | | **0 (0%)** | **7 (19%)** | **1 (3%)** | **24 (65%)** | **0 (0%)** | **25 (68%)** |
|  | 11A (n=14) | 0 (0%) | 4 (29%) | 0 (0%) | 9 (64%) | 0 (0%) | 11 (79%) |
|  | 15B (n=15) | 0 (0%) | 1 (7%) | 1 (7%) | 13 (87%) | 0 (0%) | 12 (80%) |
|  | Other (n=8) | 0 (0%) | 2 (25%) | 0 (0%) | 2 (25%) | 0 (0%) | 2 (25%) |
| **Non-vaccine (n=152)** | | **1 (1%)** | **17 (11%)** | **8 (5%)** | **80 (53%)** | **1 (1%)** | **95 (63%)** |
|  | 7C (n=10) | 0 (0%) | 3 (30%) | 0 (0%) | 2 (20%) | 0 (0%) | 4 (40%) |
|  | 9N (n=5) | 0 (0%) | 0 (0%) | 0 (0%) | 2 (40%) | 0 (0%) | 0 (0%) |
|  | 15A (n=9) | 0 (0%) | 3 (33%) | 0 (0%) | 4 (44%) | 0 (0%) | 6 (67%) |
|  | 15C (n=6) | 0 (0%) | 1 (17%) | 0 (0%) | 5 (83%) | 0 (0%) | 6 (100%) |
|  | 16F (n=11) | 0 (0%) | 1 (9%) | 1 (9%) | 4 (36%) | 0 (0%) | 9 (82%) |
|  | 17F (n=7) | 0 (0%) | 1 (14%) | 0 (0%) | 2 (29%) | 0 (0%) | 4 (57%) |
|  | 21 (n=12) | 0 (0%) | 1 (8%) | 0 (0%) | 6 (50%) | 0 (0%) | 11 (92%) |
|  | 23A (n=7) | 0 (0%) | 0 (0%) | 0 (0%) | 3 (43%) | 0 (0%) | 3 (43%) |
|  | 23B (n=29) | 0 (0%) | 3 (10%) | 2 (7%) | 27 (93%) | 0 (0%) | 25 (86%) |
|  | 35B (n=10) | 1 (10%) | 1 (10%) | 2 (20%) | 6 (60%) | 1 (10%) | 6 (60%) |
|  | 35F (n=9) | 0 (0%) | 1 (11%) | 0 (0%) | 5 (56%) | 0 (0%) | 4 (44%) |
|  | Other (n=40) | 0 (0%) | 2 (5%)s | 3 (8%) | 15 (35%) | 0 (0%) | 19 (48%) |
| ***Serotype 6C is included as a PCV-13 serotype due to cross-protection provided by serotype 6A | | | | | | | |
| TMP-SMX, trimethoprim-sulfamethoxazole | | | | | | | |
