# Supplementary material for: Serotype epidemiology and antibiotic resistance of pneumococcal isolates colonizing infants in Botswana (2016–2019)
Source: PLoS One. 2024 May 24;19(5):e0302400. doi: 10.1371/journal.pone.0302400 (PMC11125537; doi:10.1371/journal.pone.0302400)
Supplement: S4 Table — (DOCX) [file pone.0302400.s005.docx]

| **Supplemental Table 4:** Distribution of minimum inhibitory concentrations to specific antibiotics among pneumococcal isolates by year | | | | | |
| --- | --- | --- | --- | --- | --- |
| **Antibiotic** | **Year of sample collection** | | | | **p**^†^ |
|  | **2016** | **2017** | **2018** | **2019** |  |
| Amoxicillin  (median, IQR) | 0.064  (0.032, 0.190) | 0.079  (0.032, 0.250) | 0.19  (0.047, 0.250) | 0.047  (0.030, 0.190) | 0.27 |
| Azithromycin  (median, IQR) | 0.500  (0.250, 0.750) | 0.380  (0.250, 0.500) | 0.250  (0.190, 0.380) | 0.190  (0.025, 0.250) | 0.06 |
| Ceftriaxone  (median, IQR) | 0.047  (0.023, 0.190) | 0.094  (0.023, 0.190) | 0.190  (0.047, 0.250) | 0.110  (0.043, 0.205) | 0.54 |
| Penicillin  (median, IQR) | 0.032  (0.016, 0.158) | 0.125  (0.032, 0.283) | 0.250  (0.047, 0.380) | 0.125  (0.064, 0.141) | 0.45 |
| TMP-SMX  (median, IQR) | 0.750  (0.250, 1.500) | 1.5  (0.380, 3.000) | 1.5  (0.380, 3.000) | 2.000  (0.750, 3.250) | 0.62 |
| ^†^Mann-Kendall test for trend  IQR, interquartile range; TMP-SMX, trimethoprim-sulfamethoxazole | | | | | |
